# Loneliness Among US Adults During the Early Phase of the COVID-19 Pandemic: Findings From the COVID-19 Coping Study

**DOI:** 10.1093/geroni/igab046.2709

**Published:** 2021-12-17

**Authors:** Brendan O'Shea, Jessica Finlay, Jasdeep Kler, Carly Joseph, Lindsay Kobayashi

**Affiliations:** 1 University of Michigan, Lincoln, Nebraska, United States; 2 University of Michigan, Ann Arbor, Michigan, United States; 3 Central Michigan University, Ann Arbor, Michigan, United States

## Abstract

We aimed to estimate the prevalence of loneliness and identify the key sociodemographic, employment, living, and health-related risk factors for loneliness among middle-aged and older adults during the early COVID-19 pandemic in the US, when shelter-in-place and social distancing restrictions were in place for much of the country. Data were collected from online questionnaires in the COVID-19 Coping Study, a national study of 6,938 US adults aged 55-110 years, from April 2nd through May 31st, 2020. We estimated the population-weighted prevalence of loneliness (scores of ≥6/9 on the 3-item UCLA Loneliness Scale), overall and according to sociodemographic, employment, living, and health-related factors. We used population-weighted modified Poisson regression models to estimate prevalence ratios (PRs) and 95% confidence intervals (CIs) for the associations between these factors and loneliness, adjusted for age, sex, race, ethnicity, and education. Overall, 29.5% (95% CI: 27.9%, 31.3%) of US adults aged 55-110 were considered high in loneliness in April and May, 2020. In population-weighted, adjusted models, loneliness was most frequent among those with depression, those who were divorced or separated, those who lived alone, those diagnosed with multiple comorbid conditions, and individuals who were unemployed prior to the pandemic. In conclusion, we identified subpopulations of middle-aged and older US adults that were highly affected by loneliness during a period when COVID-19 shelter-in-place orders were in place across most of the country. These insights may inform the allocation of recourses to mitigate loneliness during times of restricted activity.

